# Recognition of Double-Stranded RNA and Regulation of Interferon Pathway by Toll-Like Receptor 10

**DOI:** 10.3389/fimmu.2018.00516

**Published:** 2018-03-16

**Authors:** Suki Man-Yan Lee, Tsz-Fung Yip, Sheng Yan, Dong-Yan Jin, Hong-Li Wei, Rey-Ting Guo, Joseph Sriyal Malik Peiris

**Affiliations:** ^1^HKU-Pasteur Research Pole and Center of Influenza Research, Li Ka Shing Faculty of Medicine, School of Public Health, The University of Hong Kong, Pokfulam, Hong Kong; ^2^Li Ka Shing Faculty of Medicine, School of Biomedical Sciences, The University of Hong Kong, Pokfulam, Hong Kong; ^3^Tianjin Institute of Industrial Biotechnology, Chinese Academy of Sciences, Tianjin, China

**Keywords:** toll-like receptor, TLR10, dsRNA, nucleotide-sensing receptor, IFN, interferon regulatory factor, myeloid differentiation primary response gene 88, ligand sequestration

## Abstract

Toll-like receptor (TLR)-10 remains an orphan receptor without well-characterized ligands or functions. Here, we reveal that TLR10 is predominantly localized to endosomes and binds dsRNA *in vitro* at endosomal pH, suggesting that dsRNA is a ligand of TLR10. Recognition of dsRNA by TLR10 activates recruitment of myeloid differentiation primary response gene 88 for signal transduction and suppression of interferon regulatory factor-7 dependent type I IFN production. We also demonstrate crosstalk between TLR10 and TLR3, as they compete with each other for dsRNA binding. Our results suggest for the first time that dsRNA is a ligand for TLR10 and propose novel dual functions of TLR10 in regulating IFN signaling: first, recognition of dsRNA as a nucleotide-sensing receptor and second, sequestration of dsRNA from TLR3 to inhibit TLR3 signaling in response to dsRNA stimulation.

## Introduction

Pattern recognition receptors (PRRs) play an essential role in recognizing pathogen-associated molecular patterns (PAMPs) leading to the initiation and orchestration of innate and adaptive immune responses. Toll-like receptors (TLRs) are a major group of PRRs and their activation is known to play an important role in host defense against pathogen infection (1). Ten TLR members, TLR 1–10, have been identified in humans and are responsible for the sensing of distinct microbial components. In general, TLR3, 7, 8, and 9, which are predominately located in endosomes, are involved in the recognition of nucleic acids derived from or associated with internalized microbes, while TLR1, 2, 4, 5, and 6 are localized on the surface of mammalian cells, where they can detect the outer membrane components of bacteria, fungi, and protozoan micro-organisms. Thus, the cellular localization of TLRs correlates with their functions in sensing invading pathogens. Engagement of TLRs by PAMPs leads to signaling *via* their toll/interleukin-1 (IL-1) receptor (TIR) domain recruiting signaling adaptors, and activating transcription factors that result in induction of IFNs and cytokines.

Toll-like receptor-10 is the least characterized TLR and still remains an orphan receptor, with only very limited information available regarding its localization, agonist, signaling, and function (2). A major constraint for research on TLR10 has been the lack of a suitable mouse model as TLR10 is a pseudo-gene in mice (3).

Genetic polymorphisms of TLR10 in humans are associated with autoimmune, immune-mediated diseases, viral diseases, and cancers including thyroid disease (4), Graves’ disease (5), Crohn’s disease (6), asthma (7, 8), chronic gastritis (9), complicated skin and skin structure infections (10), Crimean Congo hemorrhagic fever disease (11), urothelial bladder cancer (12), and nasopharyngeal cancer (13). TLR10 expression is also reported to be induced in response to reactive oxygen species in hypoxic cells (14). While TLR10 is implicated in the pathogenesis of several diseases, the mechanisms remain obscure.

One suggestion is that TLR10 cooperates with TLR2 in sensing bacterial lipopeptides and recruits the adaptor myeloid differentiation primary response gene 88 (MyD88) to the activated receptor complex (15). However, native TLR10 co-expressed with TLR2 as a heterodimer in a human colonic epithelial cell line did not respond to lipopeptide stimulation and a response could only be demonstrated in a situation when TLR2 was co-expressed with a chimeric TLR1/TLR10 receptor (the extracellular and transmembrane domains of TLR10, the TIR of TLR1) (15). Moreover, TLR10, alone or in cooperation with TLR2, failed to activate typical TLR-induced signaling, including activation of nuclear factor κB (NF-κB) (15). On the other hand, TLR10 has been shown to mediate activation of NF-κB and trigger innate immune responses to *Helicobacter pylori* infection (16), to act as a PRR with mainly anti-inflammatory properties inhibiting the production of pro-inflammatory cytokines in response to bacterial lipopeptides (17) and function as a negative regulator of MyD88 dependent and independent TLR signaling (18). Conversely, TLR10 may play a role in activating inflammatory responses to *Listeria monocytogenes* in intestinal epithelial cells and macrophages (19). Knockdown (KD) of TLR10 reduced TLR ligand induced pro-inflammatory cytokine expression (20) and we previously reported that TLR10 plays a role in innate cytokine responses following influenza viral infection (21). These data suggest that the modulatory effects of TLR10 are complex of which TLR10 may function distinctively in response to stimulations by different pathogens or ligands triggering distinct TLR10 signaling pathways or possibly *via* crosstalking with other PRRs.

In this study, we provide different lines of evidences demonstrating that dsRNA is a ligand for TLR10 sensing and signaling, and suggest a role of TLR10 as a nucleotide-sensing receptor. We also revealed another function of TLR10, which sequesters dsRNA from TLR3 to regulate IFN signaling. Together, these findings provide new insights into the mechanism and role of TLR10 in the regulation of IFN signaling in innate immune response.

## Materials and Methods

### Cells

THP-1 (ATCC TIB-202) cells were obtained from the ATCC and cultured in RPMI-1640 (Life Technologies) supplemented with 10% fetal bovine serum (FBS, Life Technologies), 100 U/ml penicillin and 100 µg/ml streptomycin (Life Technologies). The TLR10 KD and TLR10 overexpressed (OE) THP-1 cells were generated and maintained as described previously (21). KD and overexpression of *TLR10* was verified by RT-qPCR. THP-1-dual reporter cells were obtained from InvivoGen and maintained in RPMI-1640 culture medium with 10% FBS supplemented with 100 U/ml penicillin, 100 µg/ml streptomycin, 10 µg/ml blasticidin (InvivoGen), and 100 µg/ml Zeocin (InvivoGen). Human peripheral blood monocytes were isolated from blood packs of healthy donors provided by the Hong Kong Red Cross Blood Transfusion Service and purified by adherence and differentiated into macrophages as described (21). Consent from blood donors was obtained by Hong Kong Red Cross to use blood components for research experiments. The work involved the use of human blood samples has been reviewed and obtained human ethics approval (ref no. UW 10-201, UW 14-170) issued by Institutional Review Board of the University of Hong Kong and met the standards of the Declaration of Helsinki.

### Reagents and Antibodies

Biotin-poly(I:C) (average size: 1.5–8.0 kb), poly(I:C) (average size: 0.2–1.0 kb), and rhodamine-poly(I:C) (average size: 0.2–1.0 kb) were obtained from InvivoGen. Protein G-agarose (P-7770) (Sigma) was prepared according to the manufacturer’s instructions.

Mouse anti-human RAB5 (ab50523), mouse anti-human RAB7 (ab50533), rabbit anti-human RAB11A (ab65200), mouse anti-PDI (ab2792), mouse anti-human GIANTIN (ab37266), Alexa Fluor 555-conjugated goat anti-rabbit (ab150090), Alexa Fluor 647-conjugated goat anti-mouse (ab150115), and normal goat serum (ab7481) (all from Abcam); rabbit anti-human TLR10 (sc-30198; Santa Cruz Biotechnology); mouse anti-human TLR10 (H00081793-M01; Abnova); mouse anti-MyD88 (MA5-16231, ThermoFisher Scientific); FITC-conjugated goat anti-rabbit (F2765), Alexa Fluor 488-conjugated wheat germ agglutinin (WGA) (W11261) and NucBlue Fixed Cell ReadyProbes Reagent (4′,6-Diamidin-2-phenylindol; R37606) (all from Life Technologies) were used for immunofluorescence staining.

Mouse anti-human TLR10 (H00081793-M01, Abnova), rabbit anti-human TLR10 (sc-30198), mouse anti-β-ACTIN (MA5-15739; Invitrogen), mouse anti-MyD88 (MA5-16231; ThermoFisher Scientific), rabbit anti-TRIF (4596; Cell Signaling Technology), rabbit anti-phospho-interferon regulatory factor (IRF)-7 (Ser477) (12390, Cell Signaling Technology), rabbit anti-phospho-IRF3 (Ser396) (4947, Cell Signaling Technology), rabbit anti-human TLR3 (6961, Cell Signaling Technologies), HRP-conjugated goat anti-mouse IgG (sc-2005, Santa Cruz Biotechnology), and HRP-conjugated goat anti-rabbit IgG (sc-2004, Santa Cruz Biotechnology) were used for Western blotting. The cOmplete protease inhibitor cocktail and PhosSTOP were from Roche.

### Immunofluorescence Confocal Microscopy

THP-1 cells were washed twice with PBS and fixed with 2% paraformaldehyde (USB Corporation) in PBS for 10 min at room temperature. For the co-localization study of rhodamine-poly(I:C), TLR10 and endosomal markers, wild-type (WT) THP-1 cells and primary human monocyte-derived macrophages (MDM) were first transfected with rhodamine-poly(I:C) (10 µg/ml) for 10 and 30 min, respectively, and washed twice with PBS before fixation. For intracellular staining, cells were permeabilized using 1% saponin (Sigma) in PBS for 30 min. 0.1% saponin was included in all subsequent steps involving intracellular staining. Blocking was done by using 10% normal goat serum (Abcam) in PBS for 30 min. Cells were stained with primary and secondary antibodies in 1% normal goat serum, both for 1 h. Plasma membranes of non-permeabilized cells were stained using Alexa Fluor 488-conjugated WGA for 10 min. Nuclei were counter-stained with NucBlue Fixed Cell ReadyProbes Reagent for 5 min. Cells were embedded in Mowiol 4–88 (Sigma) with PPD (2 mg/ml) (Sigma) and 0.02% NaN_3_ (Sigma), mounted on slides with coverslips for imaging. Images were acquired using an LSM 710 (Carl Zeiss) equipped with a Plan-Apochromat 40× objective (Carl Zeiss) and processed using ZEN lite (Carl Zeiss) and ImageJ. For co-localization estimation between TLR10 and different organelles, masks were generated from separate channels from the same micrograph corresponding to TLR10 and the organelle marker. Masks were overlaid and percentage co-localization was calculated by the ImageJ algorithm.

### Ligand Stimulation

The WT, TLR10 OE, and KD THP-1 cells were stimulated with poly(I:C) (10 µg/ml or as specified), or 5′pppdsRNA (10 µg/ml) synthesized *in vitro* from vesicular stomatitis virus genome and its variant (22) for 4 h or as indicated. For intracellular stimulation, ligands were complexed with Lipofectamine 2000 reagent (Life Technologies) and delivered intracellularly. For cell surface stimulation with poly(I:C), the ligand was added to the cell culture medium directly. The induction of cytokines was analyzed by RT-qPCR (normalized to β*-ACTIN*) and compared with the corresponding unstimulated control cells.

### *In Vitro* Binding Assay

Binding assays were performed as described (23) with minor modifications. THP-1 WT cells were lysed in Buffer L (50 mM Tris–Cl, 150 mM NaCl, 5 mM EDTA, 1% IGEPAL CA-630, pH 5.5, and Roche cOmplete protease inhibitor cocktail) or 1% IGEPAL CA-630 buffer (50 mM Tris–Cl, 150 mM NaCl, 5 mM EDTA, 1% IGEPAL CA-630, pH 7.4, and Roche cOmplete protease inhibitor cocktail). Lysate was clarified by centrifugation at 12,500 × *g* for 5 min. Biotin-labeled poly(I:C) (used at 5 ng/ml, average size: 1.5–8.0 kb) was added to the lysate with or without addition of unlabeled competitive poly(I:C) (50 ng/ml, average size: 1.5–8.0 kb). Mixtures were incubated at 37°C for 1 h under gentle rotation. Cell lysates were then mixed with streptavidin agarose beads (ThermoFisher Scientific) for 2 h at 4°C. After incubation, beads were thrice washed with ice-cold PBS at the experimental pH, eluted by heating at 95°C with 2× Laemmli buffer for 10 min and analyzed by Western blotting.

### Fluorescent Resonance Energy Transfer (FRET) Assay

Interactions between poly(I:C) and TLR10 within cells were analyzed by FRET from FITC-conjugated antibodies to rhodamine-labeled poly(I:C). Rhodamine-labeled poly(I:C) was transfected into WT THP-1 cells, washed, and fixed at 30 min post-transfection as described above. Immunostaining was performed as above. Samples were mounted on slides with Mowiol 4–88 (Sigma) with PPD (1 mg/ml) (Sigma) and 0.02% NaN_3_ (Sigma). FITC and rhodamine were excited with 488 and 561 nm lasers, respectively. Images from the FITC and rhodamine channels were acquired with LSM 710 (Carl Zeiss) before, between and after repeated bleaching of rhodamine by a 561 nm laser. FRET efficiency was estimated with ZEN 2012 equipped with the FRET module using the acceptor photobleaching approach.

### Protein Sequence Alignment

The alignment of protein sequences of TIR domains of TLRs were performed using Clustal Omega (http://www.ebi.ac.uk/Tools/msa/clustalo/). The protein sequences of TIR domains were acquired from UniProt. UniProt entry identifiers for the sequences were listed in Table 1. Conserved motif was identified using MEME Suite (http://meme-suite.org/tools/meme).

**Table 1:** Toll-like receptor (TLR) protein sequences UniProt entry identifiers.

Protein	Entry identifier
TLR1	Q6FI64
TLR2	O60603
TLR3	O15455
TLR4	O00206
TLR5	O60602
TLR6	Q9Y2C9
TLR7	Q9NYK1
TLR8	Q9NR97
TLR9	Q9NR96
TLR10	D1CS19

### Immunoprecipitation

THP-1 cells were stimulated with poly(I:C) by transfection as described above. Cells were washed with ice-cold PBS and lysed in lysis buffer (50 mM Tris, 150 mM NaCl, 0.5% IGEPAL CA-630, pH 7.5, and Roche cOmplete protease inhibitor cocktail). The lysates were cleared by centrifugation at 12,000 rpm at 4°C for 15 min. Supernatants were collected and incubated with an anti-TLR10 antibody (sc-30198) for 2.5 h at 4°C. Subsequently, Protein G Agarose beads were added to the complex and incubated at 4°C for 2.5 h. Beads were washed thrice with ice-cold PBS and bound proteins were eluted by heating with 2× Laemmli buffer at 95°C for 10 min.

### Western Blotting

Cells were washed and whole cell lysates were harvested as described above. PhosSTOP was included in lysis buffer for samples immunoblotted for phosphorylated IRFs. Bands were detected with the antibodies stated and developed with Amersham ECL Prime Western Blotting Detection Reagent (GE Healthcare). Chemiluminescent signals were captured by ImageQuant LAS 4000 mini (GE Healthcare). Images acquired were analyzed with ImageQuant TL 1D version 8.1.0.0 (GE Healthcare).

### Small Interfering RNA (siRNA) Gene KD

Small interfering RNA against *TLR3* (Accell Human TLR3 7098) and/or siRNA against *TLR10* (Accell Human TLR10 81793 siRNA) or non-targeting control siRNAs (all from GE Dharmacon) were added to THP-1 cells or THP-1-Dual reporter cells at 3 µM for 72 h according to manufacturer’s protocol.

### IRF Luciferase Activity Assay

THP-1-dual reporter cells were stably integrated with two inducible reporter constructs allowing the activation of the NF-kB or IRF pathways to be detected *via* measurement of secreted alkaline phosphatase or luciferase activity, respectively. At 24 h after poly(I:C) (10 µg/ml) stimulation, secreted luciferase in supernatant from the reporter cells was quantified by the QUANTI-Luc assay (InvivoGen) using a MicroBeta luminescence counter (PerkinElmer Wallac).

### Synthesis of hTLR10-Ectodomain (ECD)

Plasmid pcDNA3-*TLR10*-YFP (Addgene 13643) was a gift from Doug Golenbock and used as a template to construct the extracellular domain of human TLR10 (hTLR10-ECD). The gene fragment of hTLR10-ECD without signal peptide was amplified by PCR using forward primer 5′-GACGACGACAAGATGGATGCTCCAGAGCTGCCAG-3′ and reverse primer 5′-GAGGAGAAGCCCGGttaTGTGTTGCAAGATAATTCGTGG-3′. The amplified gene was cloned into pET46 EK/LIC vector based on commercial provided protocol (Novagen), and the sequence of resulting plasmid pET46/hTLR10-ECD was confirmed by sequencing (Invitrogen). Recombinant hTLR10-ECD was synthesized and the protein purification procedure was carried out at 4°C as following described. The pET46/hTLR10-ECD was transformed into *Escherichia coli* BL21(DE3) cells, and single colony was selected and inoculated into 100 ml of LB containing 100 µg/ml ampicillin and culture for overnight at 37°C. Afterward, the culture was transferred to auto-induction medium by the ratio of 1:50 (1% tryptone, 0.5% yeast extract, 2 mM MgSO_4_, 0.5% glycerol, 0.05% glucose, 0.2% lactose, in PBS buffer, containing 100 µg/ml ampicillin). The culture was incubated at 16°C for 60 h under constant shaking at 200 rpm. Cells were harvested by centrifugation at 5,000 × g for 20 min then re-suspended in lysis buffer (25 mM Na_2_HPO_4_, 25 mM KH_2_PO_4_, 350 mM NaCl, 10 mM imidazole, and 5% glycerol, pH 7.2), followed by disruption with French Press. Cell debris was removed by centrifugation at 17,000 × g for 1 h. The supernatant was then applied to a Ni-NTA column using the FPLC system (GE Healthcare). The target proteins eluted at ~100 mM imidazole when using a 10–500 mM imidazole gradient. Protein-containing fractions were collected and dialyzed against a buffer containing 25 mM Tris–HCl and 20% glycerol (pH 7.5). The protein was then passed through a DEAE column and target fractions were collected and concentrated. The protein solution was then applied onto a size exclusion column (Superdex 200 16/60, 120 ml, GE HealthCare). The purified protein was concentrated and in a buffer containing 25 mM Tris–HCl, 500 mM NaCl (pH 7.5), and the purity and molecular weight of the protein were checked by SDS-PAGE.

### dsRNA Competition by TLR10 and TLR3 ECD

In-house synthesized TLR10-ECD (corresponds to residues 20–576 of a reference sequence NCBI accession number AAY78486.1) or commercially available TLR3 ECD (ab73825, Abcam) was used at a concentration of 61.9 nM and incubate with 50 ng biotin-poly(I:C) (average size: 1.5–8.0 kb; final concentration: 1 µg/ml) in PBS at pH 5.5 at 37°C in a volume of 50 µl for 1 h with gentle mixing at 15 min intervals. After incubation, mixture was further incubated with 10 µl agarose streptavidin at 4°C for 16 h. Beads were washed thrice with ice-cold PBS at pH 5.5 supplemented with 0.05% Tween-20 and eluted by heating with 2× Laemmli buffer at 95°C for 10 min. For TLR10 and TLR3 ECD competition, binding volume and elution volume were increased to twice of those set-up which individual TLR ECD was used, while keeping the concentration of both TLR ECD and biotin-poly(I:C) as well as elute gel loading volume for Western blotting analysis constant.

### RT-qPCR

Cells were lysed with RLT lysis buffer and total RNA was isolated using the RNeasy mini kit according to manufacturer’s instructions (QIAGEN). Following quantification by NanoDrop, 1 µg of RNA from each sample was used for reverse transcription using SuperScript VILO (Life Technologies). Gene expression levels were monitored using the SYBR Fast qPCR master mix kit (KAPA Biosystems) with the use of specific primers and signals were detected by a Light Cycler LC480 Instrument II (Roche). Fold change of target gene expression level was determined by the 2^−ΔΔCT^ method by utilizing β*-ACTIN* gene expression level as the internal reference.

### Statistical Analysis

All statistical analyzes were performed using an unpaired two-tailed Student’s *t*-test using GraphPad Prism 6.01 software. Data are presented as mean with SEM. *p*-Values <0.05 were considered as statistically significant.

## Results

### Sub-Cellular Localization of TLR10

**Figure 1:**
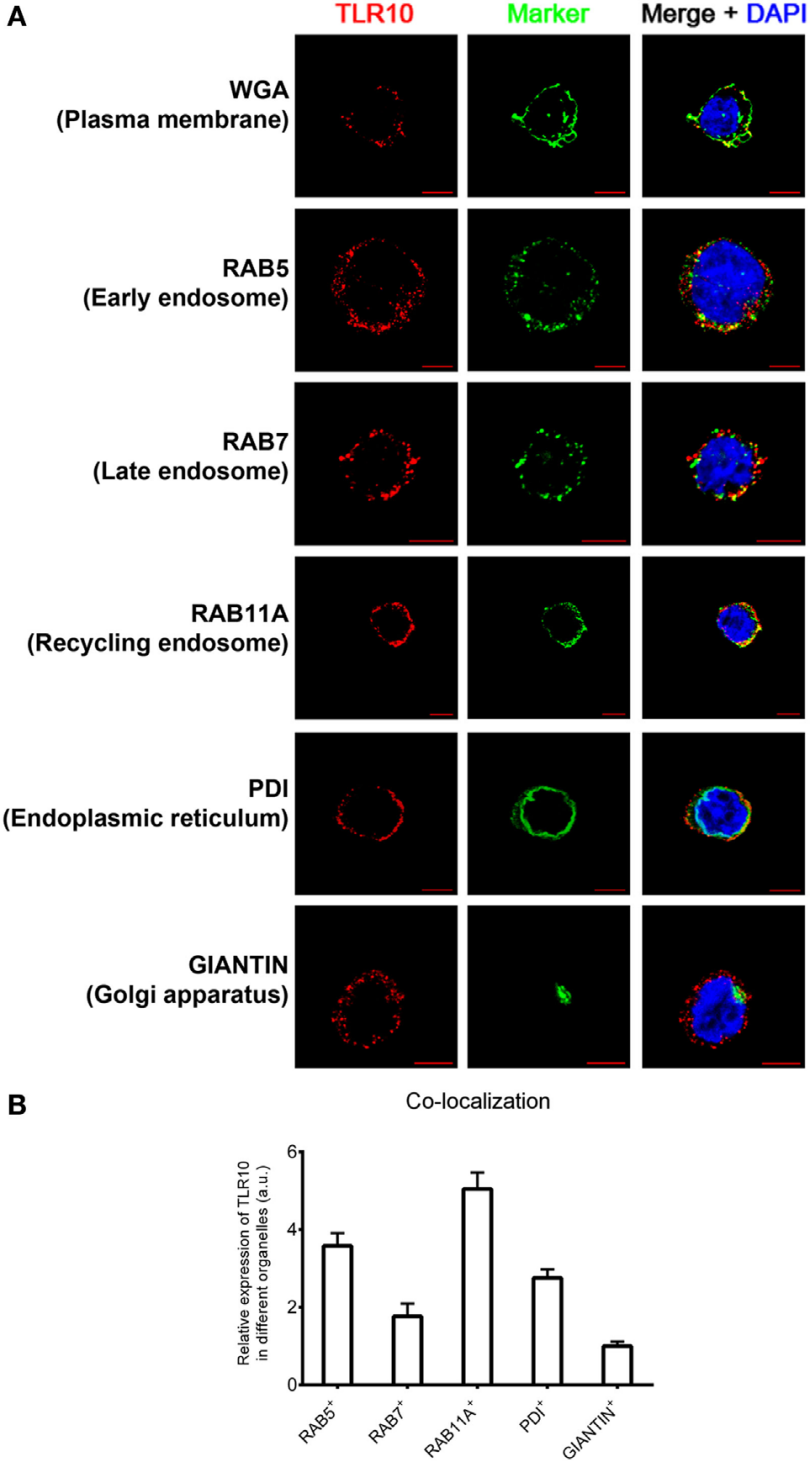
Sub-cellular localization of toll-like receptor (TLR)-10 in THP-1 cells. **(A)** Confocal micrograph of resting wild-type THP-1 cells stained for TLR10 (red), organelle markers (green), and nuclei (blue) stained with DNA-binding dye 4′,6-Diamidin-2-phenylindol (DAPI). Co-localization of TLR10 and respective organelle marker (yellow). Scale bars, 5 µm. **(B)** Relative expression of TLR10 in different organelles. The expression level of TLR10 in different organelles was compared with those expressed in the Golgi apparatus (GIANTIN^+^). Signals of more than 30 randomly picked cells from three independent experiments were computed using ImageJ with the co-localization plug-in. Data are presented as mean with SEM.

The cellular localization of a TLR has a key influence on its functions in sensing ligands (24, 25). Here, confocal microscopy was used to define the sub-cellular localization of TLR10. As in our previous flow cytometry study of a resting monocytic cell line (THP-1) (21), TLR10 was detected on cell surface but was more abundant intracellularly (Figure 1A). Markers of intracellular organelles were used to investigate the co-localization of TLR10 in different cellular compartments. TLR10 was predominately expressed in endosomes, with the highest expression detected in RAB11A^+^ recycling endosomes and RAB5^+^ early endosomes (Figure 1B). The expression level of TLR10 was high in the endoplasmic reticulum and RAB7^+^ late endosomes but relatively lower in the Golgi apparatus.

Sub-cellular localization of TLR10 in primary human MDM was also investigated (Figure S1 in Supplementary Material). As in THP-1 cells, TLR10 was detected in different organelles with a high co-localization of TLR10 with endosomal markers in MDM. This suggests that distribution of TLR10 in human THP-1 cell line closely resembles primary human macrophages. As genetic manipulation of these cells is needed for functional studies, the THP-1 cell line was used in most of the subsequent experiments.

To confirm the specificity of anti-human TLR10 antibodies used in this study, we have tested the antibodies used for Western blotting and immunofluorescence staining in WT and TLR10 genetic modified THP-1 cells. In Western blotting, protein expression level of TLR10 was found to correlate well in these cell types, of which TLR10 protein was found to be more and less abundant in TLR10 OE and KD cells, respectively, compared with WT cells (Figure S2A in Supplementary Material). Immunofluorescence staining data were also in agreement with Western blotting data, and expression in TLR10 OE cells showed stronger intensity compared with that of WT cells. Particularly, there was a significant differential intracellular expression in TLR10 OE vs WT cells (Figure S2B in Supplementary Material). These data confirmed the quality and specificity of the antibodies used in this study.

### dsRNA Is a Ligand for TLR10 Sensing and Signaling

Toll-like receptor-10 has been demonstrated to play a role in response to different immunological stimulations (10, 16–21), however, none of these studies provided evidences to demonstrate what could be the true ligand(s) for TLR10 sensing and signaling. Our finding of high-TLR10 expression in early endosomes of resting cells suggested that TLR10 might be a nucleic acid sensing receptor (23, 26). While poly(I:C) is the only nucleic acid candidate so far reported to trigger TLR10-dependent signaling (18), no previous data have shown that poly(I:C) could bind TLR10 as its ligand. Thus, we investigated if poly(I:C) is a ligand for TLR10 as well as mechanism for its signaling.

**Figure 2:**
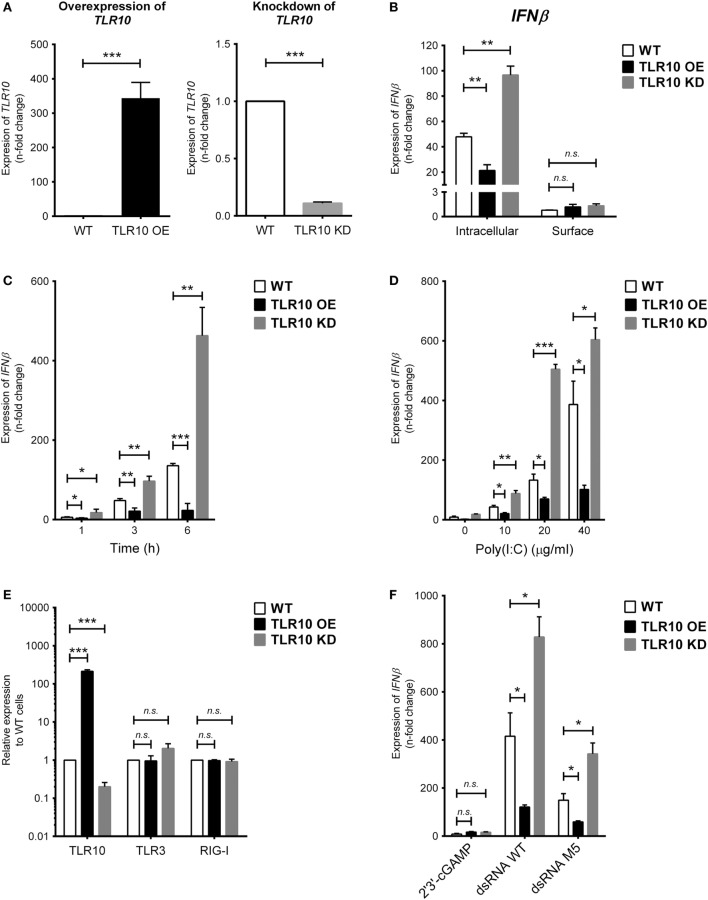
Toll-like receptor (TLR)-10 regulates dsRNA-mediated type I IFN expression. **(A)** Basal expression of *TLR10* in unstimulated wild-type (WT), TLR10 overexpressed (OE), and knockdown (KD) THP-1 cells. **(B)** Expression of *IFN*β in WT, TLR10 OE, and TLR10 KD THP-1 cells upon challenge by 10 µg/ml poly(I:C) at 4 h post-stimulation. Intracellular: poly(I:C) transfected by cationic lipid delivery; surface: poly(I:C) added to cell culture medium directly. **(C,D)** Expression of *IFN*β in WT, TLR10 OE, and KD THP-1 cells at different time points **(C)** and concentrations **(D)** upon poly(I:C) stimulation. **(E)** Basal expression of *TLR3* and *RIG-I* compared with TLR10 in WT, TLR10 OE, and KD THP-1 cells. **(F)** Expression of IFNβ in WT, TLR10 OE, and KD THP-1 cells upon stimulation by 2′3 ′-cGAMP, 5′pppdsRNA synthesized *in vitro* (dsRNA WT) or its variant (dsRNA M5). Data are mean with SEM from three independent experiments. *p < 0.05, **p < 0.01, ***p < 0.001, n.s., not significant.

To study the TLR10-specific biological function, TLR10 OE and KD cells were employed and compared with the WT THP-1 cells (21). Differential expression of TLR10 mRNA and protein in OE and KD cells was systematically checked using reverse transcription–quantitative PCR (RT-qPCR) (Figure 2A) and Western blotting (Figure S2A in Supplementary Material), respectively, and was found to be consistently maintained throughout this study. Poly(I:C) was used to stimulate these three types of cells both at the cell surface with naked poly(I:C) and transfected intracellularly *via* cationic lipid mediated delivery. When poly(I:C) was transfected, it potently induced type I IFN response in WT THP-1 cells (Figure 2B, *intracellular*). Relative to the induction of *IFNβ* in WT cells, a significantly higher level of induction occurred in TLR10 KD cells while overexpression of TLR10 reduced the IFN response (Figure 2B, *intracellular*). Naked poly(I:C) could also induce *IFNβ* expression in WT cells but to a much lesser extent compared that to transfected poly(I:C), while *IFNβ* expression in TLR10 OE or KD cells showed no difference relative to WT cells (Figure 2B, *surface*). As β*-ACTIN* was used for RT-qPCR normalization, expression of β*-ACTIN* in response to ligand stimulations was monitored. The expression of β*-ACTIN* was found to be stable, and there was no significant difference in its expression with or without ligand stimulations (Figure S2C in Supplementary Material), verifying its suitability as a house-keeping gene for RT-qPCR normalization in this study. The differential responses to intra- and extracellular stimulation of poly(I:C) has been reported, with a 1,000- to 10,000-fold increase for intracellular transfection (27, 28). These data suggest that sensing of poly(I:C) by TLR10 likely occurs in intracellular compartments and to certain extent in accordance with the abundant expression of TLR10 found intracellularly compared with that on the cell surface.

Poly(I:C) stimulates TLR10 mediated IFN response in a time- and dose-dependent manner. Changes in the expression of type I IFN among WT, OE, and KD THP-1 cells were consistent and showed a significant difference as early as at 1 h and at 3 and 6 h post-stimulation (Figure 2C) and at concentrations from 10 to 40 µg/ml (Figure 2D).

As specific features on nucleic acids have been reported to be crucial for the activation of certain PRRs (29, 30), a synthetic 5′pppdsRNA (dsRNA WT) and its variant with structural modifications and improved antiviral properties (dsRNA M5) (31) were tested for the effect of triphosphorylation at 5′ end of dsRNA on TLR10 sensing and signaling. Given that dsRNA and 5′pppdsRNA are known to sense by TLR3 and RIG-I, respectively, we first checked if basal expression of these receptors is being affected in the TLR10 OE and KD cells. Basal expression of TLR3 and RIG-I was not affected in TLR10 genetic modified cells (Figure 2E). Similar to response with poly(I:C) challenge, *IFNβ* expression is significantly induced by these 5′pppdsRNAs in WT THP-1 cells, while overexpression and KD of TLR10 suppresses and upregulates *IFNβ* expression, respectively (Figure 2F). Although expression of *IFNβ* was higher with 5′pppdsRNA, this differential expression of *IFNβ* in OE or KD cells relative to WT cells was comparable with that observed with poly(I:C), suggesting that TLR10 signaling does not preferentially sense 5′pppdsRNA over dsRNA. TLR10 regulated type I IFN expression specifically responds to dsRNA as stimulation by 2′3′-cGAMP, a ligand of the endoplasmic reticulum adaptor STING, showed a much smaller level of type I IFN induction (a 5.5-fold increase) in WT THP-1 cells and was not significantly different from that in TLR10 OE and KD THP-1 cells (Figure 2F).

Taken together, the down-regulation of the IFN response in TLR10 OE cells implies that TLR10 negatively modulates IFN responses after dsRNA stimulation.

### Binding of dsRNA to TLR10 Requires Acidic pH

**Figure 3:**
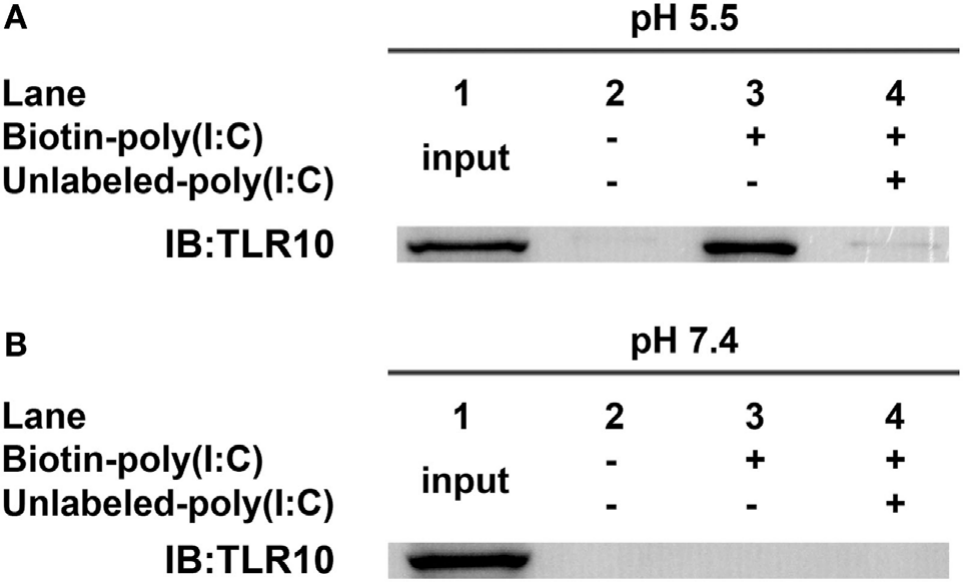
Toll-like receptor (TLR)-10 binds dsRNA *in vitro*. **(A,B)** Cell lysates of THP-1 cells were incubated with biotin-poly(I:C) (5 ng/ml) at **(A)** pH 5.5 or **(B)** pH 7.4, with or without addition of competitive unlabeled poly(I:C) (50 ng/ml) for 1 h. Complexes were pulled-down using streptavidin beads and analyzed by Western blotting using anti-TLR10 antibody. Data shown are representative of at least three independent experiments.

*In vitro* binding assays (23, 32) of TLR10 and dsRNA were performed at pH 5.5 as high-affinity ligand binding and signaling of nucleic acid sensing TLRs depends on a pH environment (33, 34) similar to that within endosomes (pH 4.5–6.5) (35–37). TLR10 was readily pulled-down *in vitro* using biotinylated poly(I:C) as bait at pH 5.5 (Figure 3A, *lane 3*). Addition of unlabeled poly(I:C) markedly decreased the amount of TLR10 pulled-down (Figure 3A, *lane 4*), suggesting that TLR10 specifically bound poly(I:C). At pH 7.4, the physiological pH (38–40) and of the cell culture and cell surface in the experiments above, TLR10 pull-down was not detectable (Figure 3B). This is consistent with the hypothesis that binding of dsRNA to TLR10 occurring in acidic compartments such as the endosomes and not at the surface of cells.

### Intracellular Interactions Between TLR10 and dsRNA Confirmed by FRET Assay

**Figure 4:**
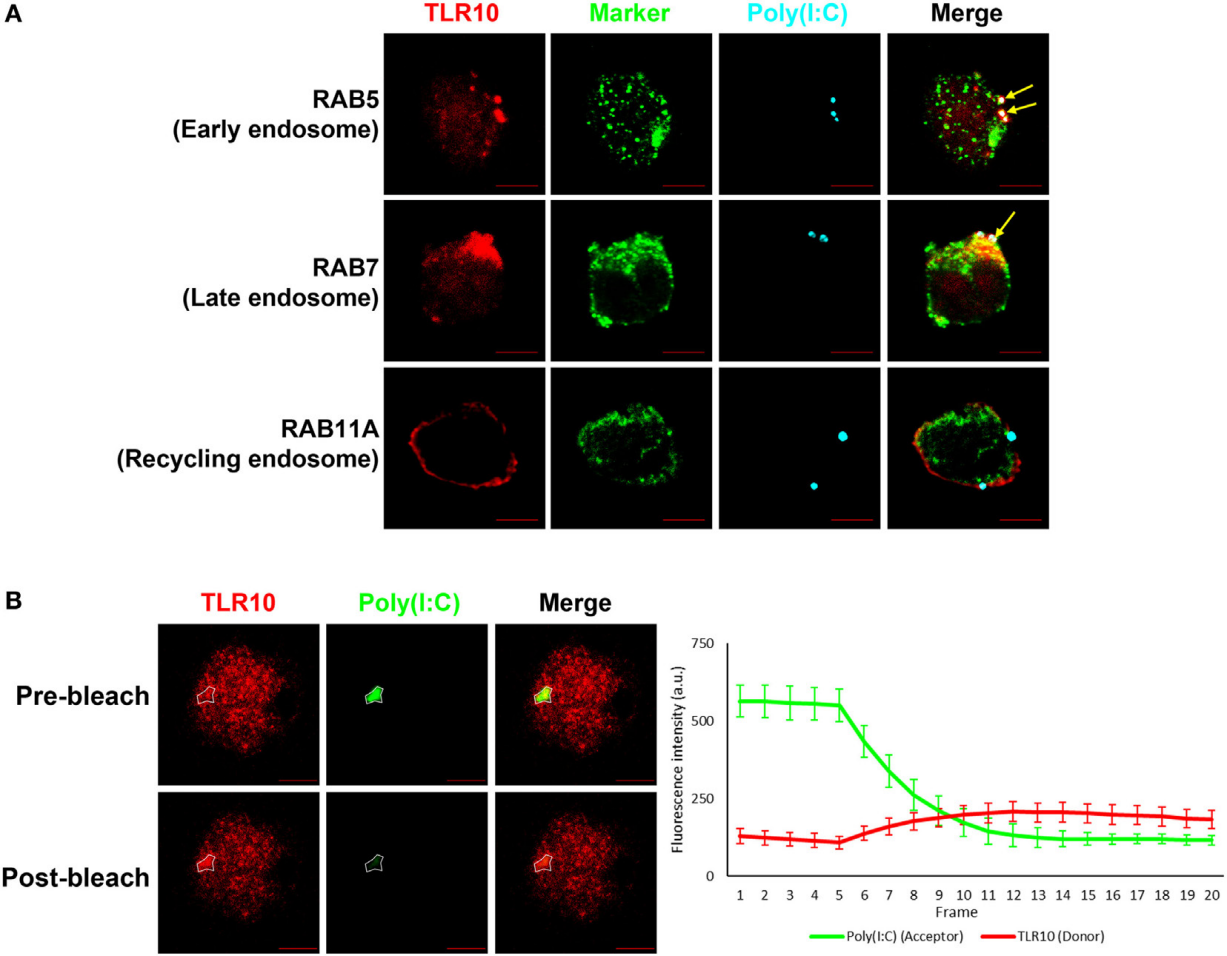
Interaction between toll-like receptor (TLR)-10 and poly(I:C) was observed by fluorescent resonance energy transfer (FRET) after acceptor photo bleaching. **(A)** Confocal micrograph of THP-1 stained TLR10 (red), organelle marker (green), and transfected fluorophore-conjugated poly(I:C) (cyan). Arrows indicate the co-localization of TLR10 and poly(I:C) in corresponding endosomal compartments (white). **(B)** Fluorophore-conjugated poly(I:C) were transfected to THP-1 cells. Channels corresponding to TLR10 (red) and poly(I:C) (green). Gradual photo-bleaching of the acceptor by 561 nm laser followed by signal capture from both channels starts after the fifth frame. Merged images depicting co-localization of TLR10 and poly(I:C) with the region of interest for acceptor and donor images before and after bleaching circled. Quantification of FRET for the circled region is displayed graphically as fluorescence intensity over frame. Scale bars, 5 µm. Data are mean with SEM of eight individual samples.

A co-localization study of the spatial association of TLR10 with poly(I:C) showed that, after ligand transfection, fluorophore-labeled poly(I:C) co-localized with TLR10 in RAB5^+^ early or RAB7^+^ late endosomes, but this was barely detectable in RAB11A^+^ recycling endosomes (Figure 4A). Similar result was observed in primary human MDM cells showing co-localization of TLR10 and poly(I:C) in endosomes (Figure S3 in Supplementary Material).

Next, we determined by FRET assay to investigate if TLR10 and poly(I:C) are sufficiently close such that they interact with each other. Fluorescently labeled poly(I:C) as FRET acceptor were transfected into THP-1 cells. Endogenous TLR10 detected by fluorophore-conjugated antibody was served as FRET donor in the assay. To better avoid artifacts which may be introduced by the single acceptor photo-bleaching approach, a gradual acceptor photo-bleaching was chosen (41). Signal from both TLR10 corresponding channel (donor) and poly(I:C) corresponding channel (acceptor) within the bleached region of interest (Figure 4B, *white circled*) was monitored in real-time throughout photo-bleaching which started after the fifth frame. After photo-bleaching of the acceptor, fluorescence intensity of the donor corresponding to TLR10 increased obviously in the bleached region with calculated mean FRET efficiency of 50.4 ± 12.7%. This result suggests that TLR10 and poly(I:C) are at very close proximity and further implies that TLR10 and dsRNA interact directly with each other.

### MyD88 Is Recruited to TLR10 Upon dsRNA Stimulation

**Figure 5:**
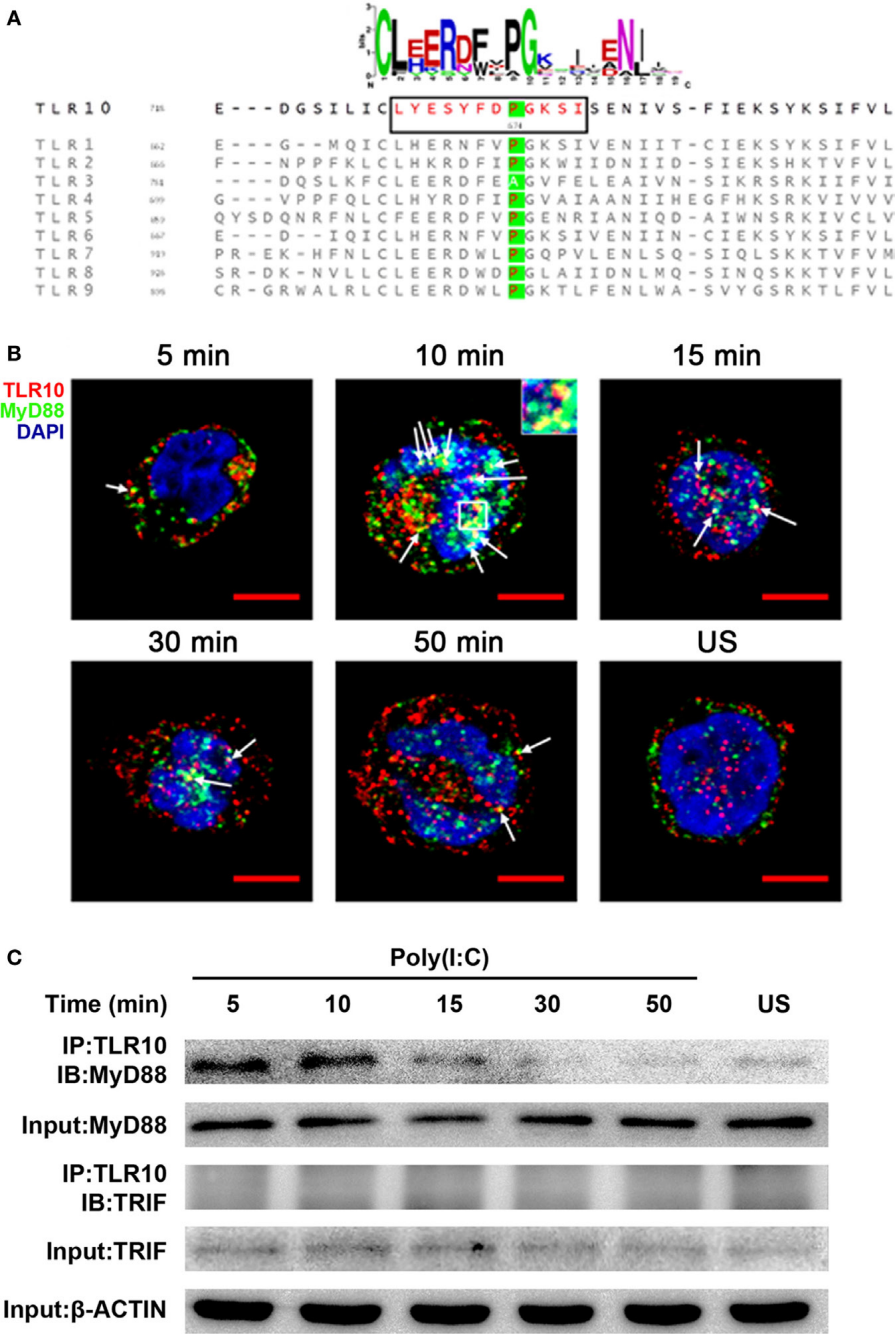
Myeloid differentiation primary response gene 88 (MyD88) is the adaptor protein for toll-like receptor (TLR)-10 signaling following stimulation by dsRNA. **(A)** Alignment of toll/interleukin-1 receptor domain sequences of human TLRs. Sequence logo (top) represents the conserved motif identified by MEME. Sequence in black box is the BB-loop sequence in TLR10. The alanine/proline residues highlighted in green determine the adaptor protein bound by TLRs. All human TLRs, except TLR3, have proline in the BB-loop. **(B)** Confocal micrograph of THP-1 cells stimulated with 10 µg/ml poly(I:C) stained at different time points for TLR10 (red), MyD88 (green), with nuclei stained with DNA-binding dye 4′,6-Diamidin-2-phenylindol (DAPI) (blue). Unstimulated (US) cells were included as a control. Arrows indicate the co-localization of TLR10 and MyD88 (yellow). Inset (at 10 min post-challenge) is an enlargement of the white square box. Scale bars, 5 µm. **(C)** TLR10 interacts with MyD88 upon poly(I:C) stimulation. Cell lysates of THP-1 cells stimulated with 10 µg/ml poly(I:C) at different time points were immunoprecipitated using anti-TLR10 antibody and then analyzed by Western blotting using anti-MyD88 or anti-TRIF antibodies. β-ACTIN was the input control. Data shown are representative of at least two independent experiments.

The highly conserved BB-loop of the TIR domain of TLRs was shown to interact with TIR domains of signal-activating adaptor proteins (24, 42–44) and an alanine/proline residue in the BB-loop confers adaptor binding specificity (42, 45). Except TLR3, all known human TLRs, including TLR10, have a proline residue in the BB-loop, and are thought to bind to MyD88 (Figure 5A). TLR3 has alanine in this position and binds TIR-domain-containing adaptor-inducing IFNβ (TRIF), yet mutation of this alanine residue to proline is sufficient to switch the TLR3 signaling adaptor from TRIF to MyD88 (45). Based on sequence similarity, MyD88 would be expected to be an adaptor protein for TLR10 signaling.

Co-localization of MyD88 with TLR10 upon poly(I:C) stimulation was investigated. Cells not transfected with poly(I:C) displayed little or no co-localization of MyD88 and TLR10 (Figure 5B). However, on transfection with poly(I:C), co-localization of MyD88 with TLR10 was observed as early as 5 min post-ligand stimulation, with increased levels at 10 min, and a subsequent decline. Recruitment of MyD88 by TLR10 upon stimulation was further confirmed by immunoprecipitation (Figure 5C). In a mock transfection [without poly(I:C)], MyD88 was barely detectable in samples immunoprecipitated with an anti-TLR10 antibody while a stronger interaction between MyD88 and TLR10 was detected upon poly(I:C) stimulation (Figure 5C). As in the co-localization study, the strongest interaction was detected at 10 min post-stimulation and decreased gradually afterward. Immunoprecipitation of TLR10 with TRIF did not result in pull-down of TRIF (Figure 5C). Thus, MyD88, but not TRIF, is actively recruited to TLR10 upon poly(I:C) stimulation.

### TLR10 Regulates dsRNA-Mediated IFN Responses *via* IRF7

**Figure 6:**
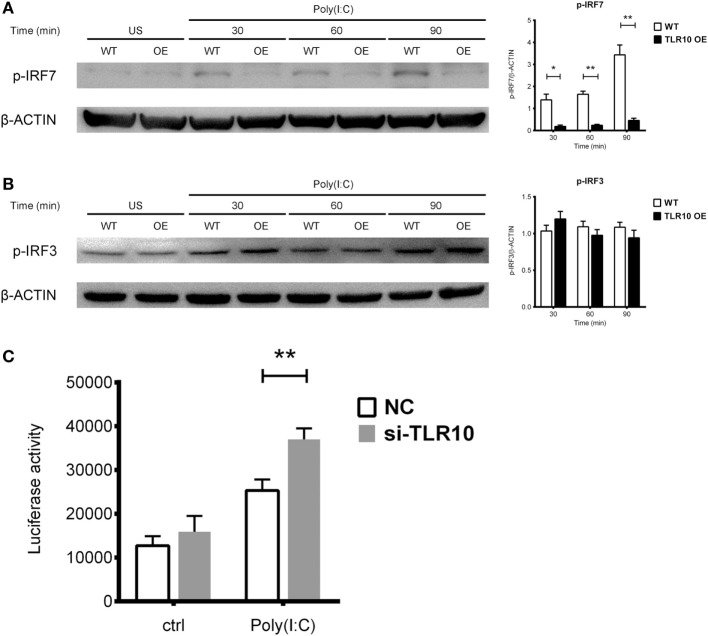
Toll-like receptor (TLR)-10 stimulated by dsRNA regulates type I IFN responses through phosphorylation of interferon regulatory factor (IRF)-7. **(A,B)** Phosphorylation level of **(A)** IRF7 and **(B)** IRF3 in wild-type (WT) and TLR10 overexpressed (OE) THP-1 cells upon stimulation by 10 µg/ml poly(I:C) at different time points post-challenge was analyzed by Western blotting with anti-phospho-IRF7 (Ser477) and anti-phospho-IRF3 (Ser396) antibodies, respectively. A representative blot (left) and mean (with SEM, right) from three independent experiments are shown. **(C)** Augmented type I IFN signaling in TLR10 knockdown THP-1 cells through an IRF-inducible luciferase reporter. Luciferase activity measured in THP-1 reporter cells upon transfection with 10 µg/ml poly(I:C) in TLR10 small interfering RNA (siRNA) (si-TLR10) or a non-targeting control siRNA (NC) treated THP-1 cells. Data are mean with SEM from at least three independent experiments. *p < 0.05, **p < 0.01.

Interferon regulatory factors play an essential role in regulating IFNs expression following TLR signaling (46). IRF3 and IRF7 are thought to regulate expression of type I IFN in response to dsRNA stimulation (46). Activation of IRF7 is characterized by phosphorylation of its C-terminus by the IKK-related kinases TBK-1 and IKK_ε_, followed by IRF dimerization and nuclear translocation (47). TLR10 signaling might reduce the phosphorylation of IRFs, subsequently leading to reduced type I IFN production. Therefore, IRF phosphorylation was examined following transfection of poly(I:C) into WT and TLR10 OE THP-1 cells. Phosphorylation of IRF7 stably increased in response to poly(I:C) challenge in WT cells, while, it was markedly reduced in TLR10 OE cells (Figure 6A). Phosphorylation of IRF3 showed no differences with respect to TLR10 overexpression, and was similar in both cell types at all times following poly(I:C) challenge (Figure 6B).

Interferon regulatory factor-3 is constitutively expressed in most cells while IRF7 can be induced in response to the activation of PRRs or type I-IFN-mediated signaling (48, 49). The mRNA levels of both *IRF7* and *IRF3* were compared between WT and TLR10 OE cells upon poly(I:C) challenge (Figure S4 in Supplementary Material). Expression of *IRF7* was upregulated in WT cells but down-regulated in TLR10 OE cells at 6 h post-poly(I:C) challenge (Figure S4A in Supplementary Material). *IRF3* expression was unchanged throughout the course of the experiment (Figure S4B in Supplementary Material).

Taken together, the data here demonstrated that TLR10 regulates type I IFN response through IRF7 but not IRF3. TLR10 signaling not only modulates IRF7 activity but also its expression.

Expression of soluble luciferase in a reporter THP-1 cell line under the control of an IRF-inducible promoter (five IFN-stimulated response elements and an ISG54 minimal promoter) allows quantification of the induction of type I IFN signaling responses through the level of luciferase activity. siRNA against TLR10 (si-TLR10) were introduced to reporter THP-1 cells to compare their luciferase activities with cells treated with non-targeting control siRNA, in response to poly(I:C) stimulation. A significant increase in luciferase activity was seen in reporter cells transfected with anti-TLR10 siRNA relative to that in cells transfected with the control siRNA, further proven that IRF mediated type I IFN response would be augmented in a TLR10 deficient environment (Figure 6C).

### TLR10 Competes With TLR3 for dsRNA Binding

In this study, we demonstrated a direct binding of poly(I:C) to TLR10, while poly(I:C) either added in the culture medium or transfected directly into the cells (34, 50, 51) is also known ligand to activate TLR3 signaling (52).

**Figure 7:**
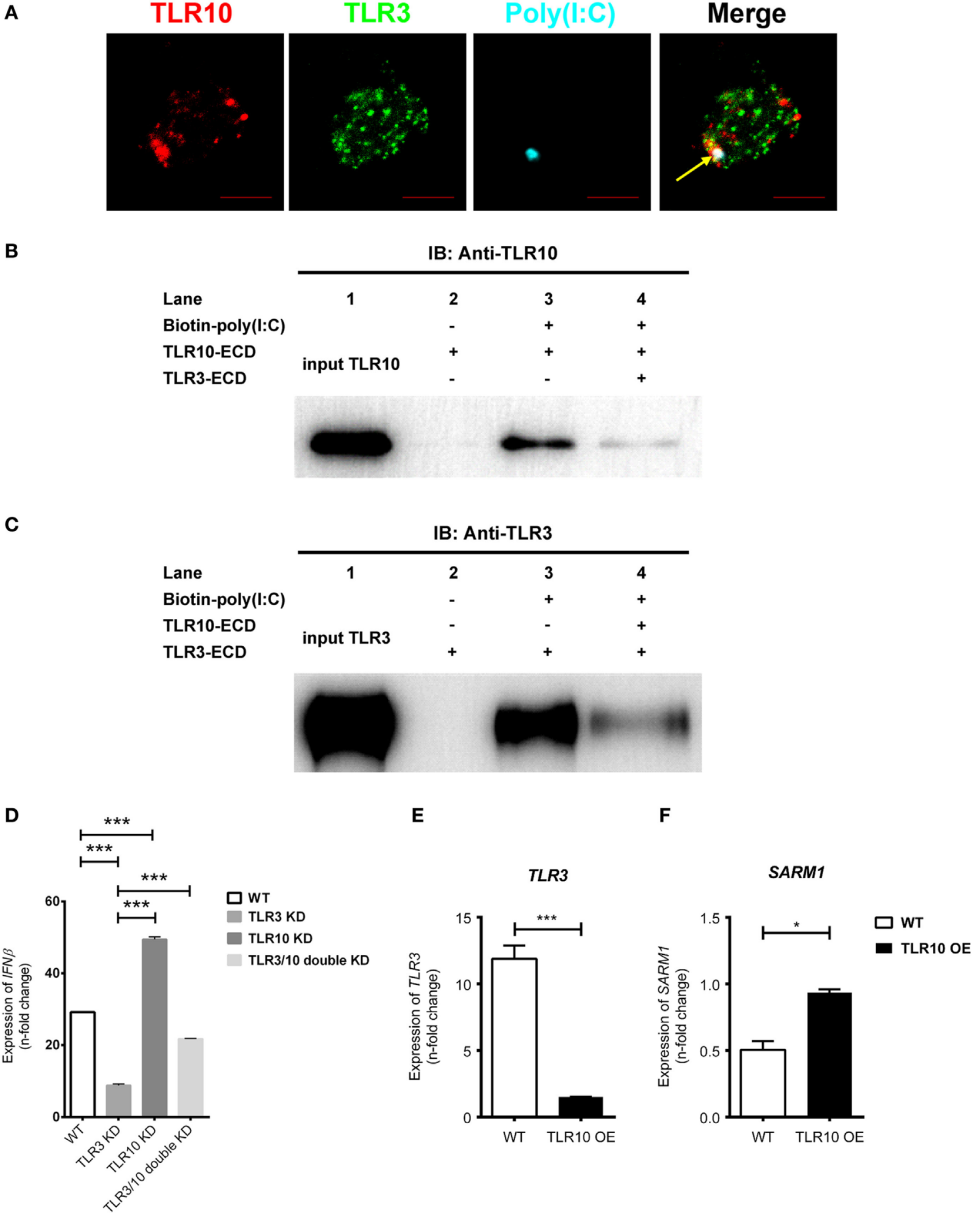
Crosstalk between toll-like receptor (TLR)-10 and TLR3. **(A)** THP-1 cells were challenged with fluorophore-conjugated poly(I:C) (cyan) and stained for TLR10 (red) and TLR3 (green). Arrow indicates co-localization of TLR10, TLR3, and poly(I:C) (white). Scale bars, 5 µm. **(B,C)** The ectodomains (ECD) of TLR10 or TLR3 recombinant proteins were incubated with biotin-conjugated poly(I:C) alone or together for 1 h at pH 5.5. The biotin-poly(I:C) bound complexes were pulled-down by streptavidin beads and analyzed by immunoblotting using anti-TLR10 **(B)** or anti-TLR3 **(C)** antibodies. **(D)** Expression of *IFN*β in wild-type (WT), *TLR3* knockdown (KD), *TLR10* KD, and *TLR3/10* double KD THP-1 cells upon poly(I:C) challenge. **(E,F)** Expression of *TLR3* **(E)** and sterile alpha and TIR motif-containing protein 1 **(F)** in WT and *TLR10* overexpressed (OE) cells in response to poly(I:C) challenge (10 µg/ml, 6 h post-stimulation). The mRNA expression was quantitated using RT-qPCR and denoted as fold change compared with corresponding unstimulated cells. Data are mean with SEM from three independent experiments. *p < 0.05, ***p < 0.001.

Since TLR3 is predominantly expressed intracellularly, ligands need to be transported into the receptor containing organelles to activate its signaling. Study demonstrated that CD14 enhances dsRNA-mediated TLR3 activation by directly binding to poly(I:C) and mediating cellular uptake of extracellular poly(I:C) (53). Ligands could be transfected into the cells with cationic liposomes of which dsRNA–liposome complexes are thought to be delivered to the endosome where they activate TLR3 (34, 50, 54, 55). Here, we found that transfected poly(I:C) co-localized together with both TLR10 and TLR3 in endosomes (Figure 7A), suggesting transfected poly(I:C) could interact with both TLRs in endosomes. A recent paper suggested that TLR10 has an inhibitory effect on IFNβ production may involve TLR3 (18), but the mechanism remains undefined. Here, we investigated the mechanism involving crosstalk between TLR10 and TLR3. We generated a recombinant TLR10 ECD to investigate ligand sequestration from TLR3. Biotinylated poly(I:C) was incubated with TLR10-ECD or TLR3-ECD alone or both together at acidic pH condition. The ECD-dsRNA complexes formed were pulled-down and analyzed by immunoblotting (Figures 7B,C). TLR10-ECD or TLR3-ECD alone could be efficiently pulled-down when they were individually incubated with biotinylated poly(I:C) (Figures 7B,C, *lane 3*), suggesting that TLR10-ECD itself, like TLR3-ECD is sufficient for dsRNA binding without the need for other protein as co-receptor *in vitro*. Notably, when TLR10-ECD and TLR3-ECD were incubated with biotinylated poly(I:C) together, the pulled-down amount of both TLR10-ECD and TLR3-ECD were markedly reduced (Figures 7B,C, *lane 4*). This suggests that TLR10 competes with TLR3 and sequester dsRNA from TLR3 binding. To further examine this, we determined the effect of TLR3/10 double KD on IFN expression. Poly(I:C) was challenged to WT, TLR3 KD, TLR10 KD, and TLR3/10 double KD THP-1 cells and expression of *IFNβ* was assayed (Figure 7D). As expected, knock-downing of TLR10 and TLR3 significantly led to up- and down- regulation of *IFNβ*, respectively, while expression of *IFNβ* was restored in TLR3/10 double KD cells similar to the level of WT cells. These data suggested that TLR3 and TLR10 have opposite effect on IFN expression, and there is a crosstalk of TLR3 and TLR10, while sequestration of dsRNA from TLR3 by TLR10 is a contributory factor to regulate dsRNA-mediated IFN response. Furthermore, when TLR10 was OE, the expression of TLR3 (Figure 7E) was significantly suppressed in response to poly(I:C) stimulation suggesting that TLR10 not only sequester ligand with TLR3, but also regulate TLR3 expression to inhibit its signaling.

We also found that Sterile alpha and TIR motif-containing protein 1 (SARM1) was reduced upon poly(I:C) challenge, while its expression was rescued with TLR10 overexpression (Figure 7F). SARM1 is a negative regulator in TLR signaling. Activation of the TRIF-dependent pathway, e.g., TLR3 signaling is suppressed by SARM1 of which SARM1 associates with TRIF and inhibits the downstream signaling (56). Here, we found that expression of *SARM1* was enhanced with TLR10 overexpression, suggesting another possible regulatory mechanism by TLR10 to suppress TRIF-dependent TLR3 signaling regulating IFN expression.

## Discussion

**Figure 8:**
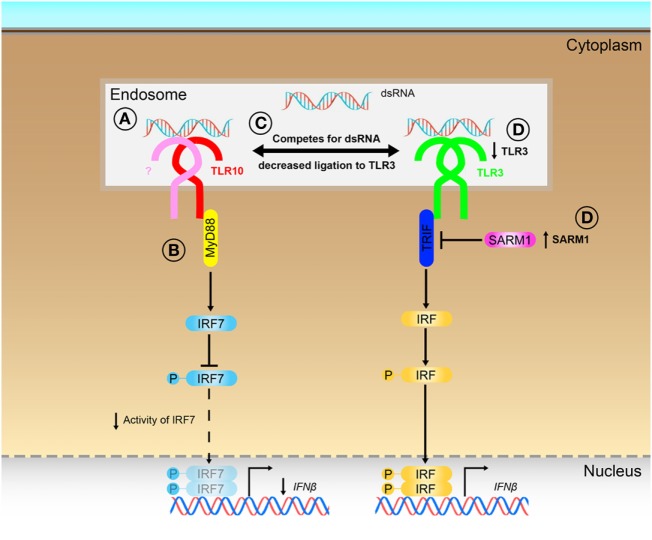
Proposed model for the novel dual functions of toll-like receptor (TLR)-10 in regulating IFN signaling. **(A)** Sensing of dsRNA by TLR10 in endosomes. If TLR10 forms homodimer/heterodimer or co-factors needed for signaling is not clear. **(B)** Activation of TLR10 recruits myeloid differentiation primary response gene 88 (MyD88), subsequently leading to decrease in interferon regulatory factor (IRF)-7 phosphorylation and suppress *IFN*β expression. **(C)** Ligand sequestration: TLR10 competes with TLR3 for dsRNA, attenuates TLR3 mediated *IFN*β expression. **(D)** Signaling of TLR10 negatively regulates TLR3 expression and promotes expression of negative regulator of the signaling, sterile alpha and TIR motif-containing protein 1 (SARM1) to further suppress TLR3 signaling and the subsequent IFNβ expression.

Although TLRs have been implicated in many diseases (4–7, 10, 12, 13), TLR10 is unique among the human TLRs in limited knowledge that exists about its ligand(s), signaling, and function. Several ligands as well as in response to bacterial and viral infections (16, 17, 19, 21), have been described to elicit a TLR10-dependent response, however, no solid evidence has been provided so far to support what could be the true ligands of TLR10 for its sensing and signaling. In this work, we provided different lines of evidences to demonstrate that dsRNA is in fact a true ligand for TLR10 sensing and signaling, thereby identifying a previously unrecognized role of TLR10 as a novel nucleotide-sensing receptor. We also revealed that TLR10 competes with TLR3 for ligand binding and proposed a model to illustrate the mechanisms for dual functions of TLR10 in the regulation of dsRNA-mediated IFN signaling (Figure 8).

To date, only nucleic acid sensing TLRs have been reported to be located in early or late endosomes in resting cells (23, 26). The expression and localization of TLR10 in RAB5^+^ early and RAB7^+^ late endosomes of resting cells is in concordance with the nucleic acid sensing nature of TLR10. While, the co-localization event of poly(I:C) and TLR10 in RAB11A^+^ recycling endosomes was barely observed. RAB11A^+^ recycling endosomes are known to transport cargo back to the plasma membrane after endocytosis (57). It is possible that TLR10 localized in RAB11A^+^ endosome would be trafficking to plasma membrane to exert its function (16, 17) and trigger same or different signaling as in intracellularly, an aspect that deserves further investigation.

Compartmentalization of nucleic acid sensing TLRs is useful to prevent aberrant activation by self-nucleic acids released from dead cells. Mis-localized TLRs have been demonstrated to be activated by self-nucleic acids and have been suggested as a cause for autoimmune diseases (58). For example, intracellular localization of TLR9 prevents recognition of self-nucleic acids but facilitates access to viral nucleic acids entering *via* the endocytic pathway. Endosomes provide acidic pH for increased affinity of nucleic acid sensing receptors to respective ligands (36, 59). TLR10 binds to dsRNA at acidic endosomal pH, it is possible that this mechanism is utilized by TLR10 as a control mechanism to minimize aberrant receptor activation.

Previous functional studies on TLR10 mainly focused on the regulation of pro-inflammatory cytokines such as IL-6 and IL-8 (10, 17, 19). A recent paper suggested the involvement of TLR10 in poly(I:C) induced IFN response, possibly *via* TLR3, suggesting a crosstalk of TLR10 with other TLR signaling pathways (18), but the mechanism remains unexplored. In our present study, we investigated on the mechanistic aspect of TLR10 biological function by demonstrating its direct ligand competition with TLR3 through sequestrating poly(I:C). Furthermore, we also propose several mechanisms in the regulation of TLR3 signaling by TLR10, *via* suppressing TLR3 expression and enhancing negative regulator SARM1 to inhibit TRIF mediated signaling.

Our data further demonstrated a novel signaling pathway of TLR10. In the canonical TLR signaling pathway, dsRNA sensed by TLR3 leads to TRIF–IRF mediated IFN response. Our data suggested that activation of TLR10 by poly(I:C) triggered the recruitment of MyD88, but not TRIF, and reduced phosphorylation of IRF7, while IRF3 was not affected. Previously, it has been shown that upon the activation of TLR9, the death domain of MyD88 could directly interact with an inhibitory domain of IRF7 but not IRF3 (60). Thus, the recruitment of MyD88 upon TLR10 stimulation demonstrated in this study suggests TLR10 mediated MyD88-IRF7 axis to regulate IFN expression. While mechanism(s) on the inhibition of IRF7 activity by TLR10 and whether if there are additional adaptors involved in such interaction deserve further investigation.

Previous studies have shown contradictory role of TLR10 either enhancing or suppressing cytokine response (16–21). Our present data demonstrated the mechanism of crosstalking between TLR10 and TLR3 put forward a new thinking to explain the divergent roles of TLR10. Involvement of TLR10 in cytokine response upon different stimulations, could be largely dependent on TLR10 signaling solely or in combination with one or more PRRs signaling. Especially in the scenario of pathogen infections, microbial components could be PAMPs to trigger a diverse array of PRRs, thus the complicated crosstalk between TLR10 and other PRRs in different disease pathogenesis would be an important area for future study. Since murine TLR10 is a pseudo-gene, investigating using classical knock-out approach is not possible in a mouse model. The generation and access of human TLR10 knock-in mice have successfully demonstrated that TLR10 has a role in controlling immune response *in vivo* (18). Our next important goal is to study the functional relevance of TLR10 as well as its crosstalk with other PRRs and their signaling employing transgenic human TLR10 knock-in mice in combination with PRR antagonist(s) or signaling inhibitor(s) in response to microbial infections.

The discovery of ligand for TLR10 is a major step in the understanding of the biological function of this hitherto orphan receptor. In this study, we provide different lines of evidences suggesting dsRNA is the ligand for TLR10. TLR10 is a novel nucleotide-sensing receptor. Our work here provides mechanistic insight explaining two major roles of TLR10 in regulating IFN response upon dsRNA stimulation: first, recognition of dsRNA as a nucleotide-sensing receptor for TLR10 signaling which involves MyD88 and IRF7 to modulate IFN response and second sequestration of dsRNA with TLR3 for inhibiting TLR3 signaling and thus IFN expression.

Results here demonstrate the mechanism underlying the crosstalk between TLR10 and TLR3, which opens up a new concept in the regulation of IFN response by TLR10. Besides ligand sequestration, sequestration of signaling molecules between TLR10 and TLR3 or other PRRs signaling will be the next step to understand the mechanistic details on such regulation.

As there is increasing evidence suggesting the involvement of TLR10 in different disease pathogenesis, we believe that these new findings not only provide important fundamental insights to the understanding of immunobiology of TLR10, but also bring indispensable importance to further investigate the role and functional relevance of TLR10 in diseases. Modulation of TLR10 signaling may thus provide a unique option to fine-tune fundamental physiological pathways involved in disease pathological conditions.

## Ethics Statement

Consent from blood donors was obtained by Hong Kong Red Cross to use blood components for research experiments. The work involved the use of human blood samples has been reviewed and obtained human ethics approval (ref no. UW 10-201, UW 14-170) issued by Institutional Review Board of the University of Hong Kong and met the standards of the Declaration of Helsinki.

## Author Contributions

SMYL and DYJ designed research. TFY, SY, HLW, and RTG performed research. SMYL, TFY, SY, and JSMP analyzed data and wrote the manuscript. SMYL and JSMP obtained research grants and provided reagents.

## Conflict of Interest Statement

The authors declare that the research was conducted in the absence of any commercial or financial relationships that could be construed as a potential conflict of interest.
