# Supplementary material for: Recognition of Double-Stranded RNA and Regulation of Interferon Pathway by Toll-Like Receptor 10
Source: Front Immunol. 2018 Mar 16;9:516. doi: 10.3389/fimmu.2018.00516 (PMC5865411; doi:10.3389/fimmu.2018.00516)
Supplement: Supplementary file 1 [file presentation_1.PDF]

Supplementary Material

Recognition of double-stranded RNA and regulation of interferon pathway by Toll-like receptor 10

Suki Man-Yan Lee[1*], Tsz-Fung Yip[1†], Sheng Yan[1†], Dong-Yan Jin[2], Hong-Li Wei[3], Rey-Ting Guo[3] and Joseph Sriyal Malik Peiris[1*]

Affiliations:

[1]HKU-Pasteur Research Pole and Center of Influenza Research, School of Public Health, Li Ka Shing Faculty of Medicine, The University of Hong Kong, Hong Kong, China

[2]School of Biomedical Sciences, Li Ka Shing Faculty of Medicine, The University of Hong Kong, Hong Kong, China

[3]Tianjin Institute of Industrial Biotechnology, Chinese Academy of Sciences, China

†These authors contribute equally

*** Correspondence:**

Dr. Suki Man-Yan Lee

suki@hku.hk

Prof. Joseph Sriyal Malik Peiris

malik@hku.hk

Supplementary Figures

Figure S1

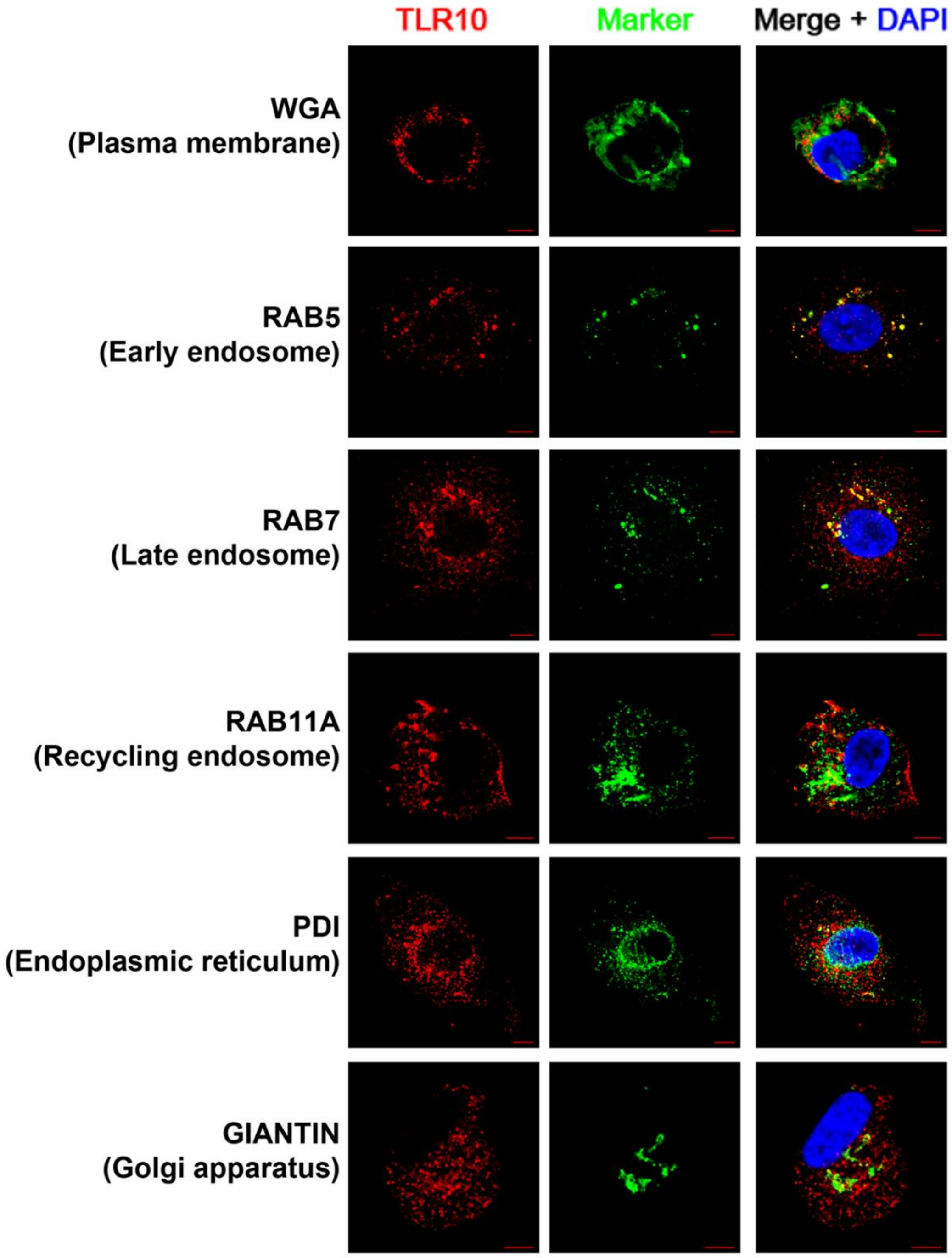

Figure S1. Sub-cellular localization of TLR10 in primary human monocyte-derived macrophages. Confocal micrograph of resting primary human monocyte-derived macrophages stained for TLR10 (red), respective organelle markers (green) with nuclei stained with DNA-binding dye DAPI (blue). Co-localization of TLR10 and respective organelle marker (yellow). Scale bars, 5 μm.

Figure S2

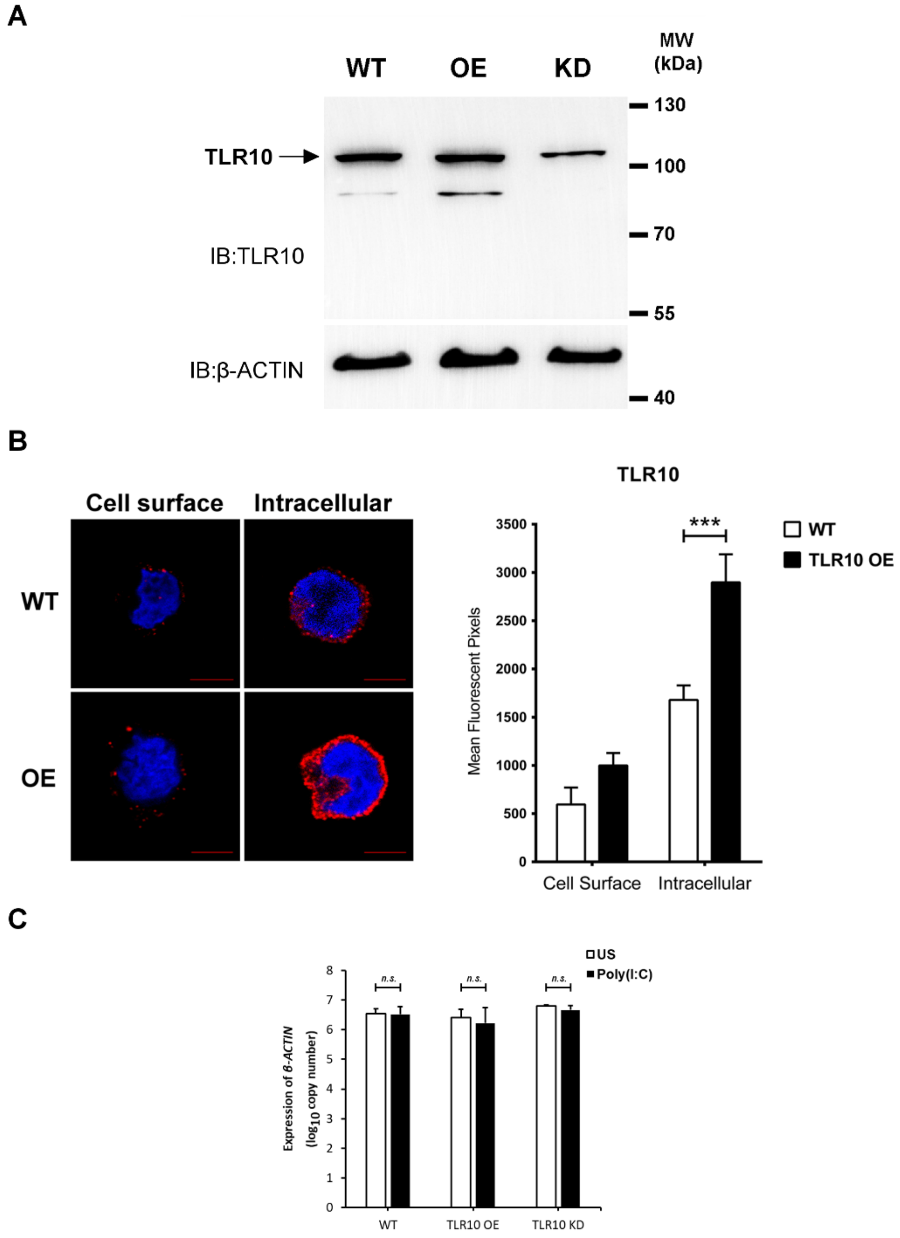

Figure S2. Quality check of anti-TLR10 antibodies using WT and TLR10 genetic modified THP-1 cells. **(A)** Expression of TLR10 protein in WT and different TLR10 genetic modified THP-1 cells was assayed by Western blotting. β-ACTIN was included as input control. **(B)** Expression of TLR10 in different cell lines assayed by immunofluorescent staining. Representative micrographs are shown on left. TLR10 (red), nuclei (blue). Scale bars, 5 μm. TLR10 expression on cell surface or intracellular in WT and TLR10 OE cells expressed as mean fluorescence pixels following immunofluorescence staining was estimated by ImageJ plugin (right). **(C)** Expression of *β-ACTIN* in WT and TLR10 genetic modified THP-1 cells in response to ligand stimulation. Data are mean with SEM from at least 3 independent experiments. ***$p < 0.001$, *n.s.* not significant.

Figure S3

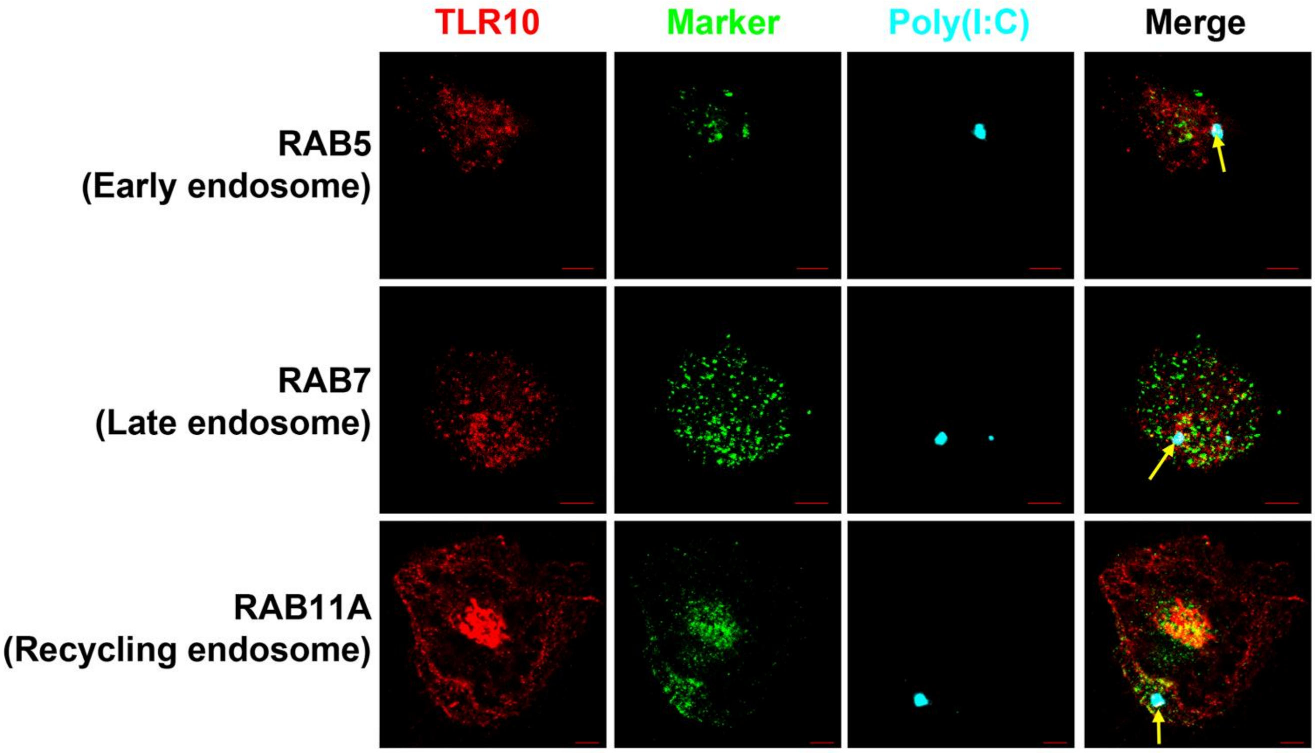

Figure S3. Co-localization of poly(I:C) and TLR10 in endosomes of primary human monocyte-derived macrophages. Confocal micrograph of primary human monocyte-derived macrophages transfected with fluorophore-conjugated poly(I:C) (cyan). Cells stained for TLR10 (red) and organelle markers (green). Arrows indicate the co-localization of TLR10 and poly(I:C) in endosomes (white). Scale bars, 5 μm.

Figure S4

A

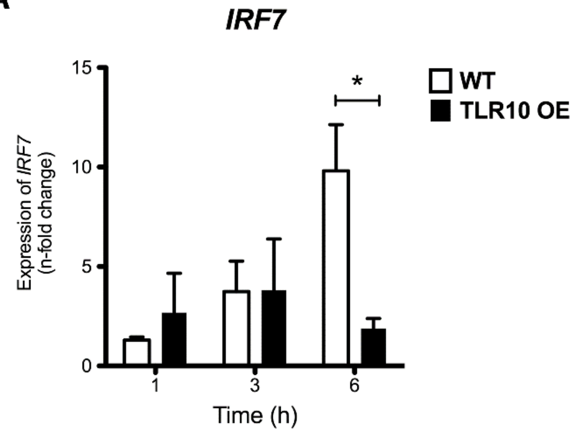

B

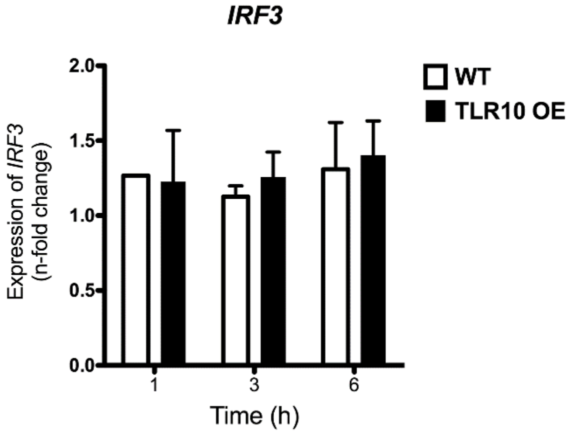

Figure S4. Expression of *IRF7* and *IRF3* in poly(I:C) stimulated cells. Expression of **(A)** *IRF7* and **(B)** *IRF3* in WT and TLR10 OE THP-1 cells transfected, at different time points, with 10 µg/ml poly(I:C) determined by RT-qPCR relative to unstimulated cells. Data shown are presented as mean fold change with SEM from at least three independent experiments. *$p < 0.05$.
